# Supplementary figures and images for: College student Fear of Missing Out (FoMO) and maladaptive behavior: Traditional statistical modeling and predictive analysis using machine learning
Source: PLoS One. 2022 Oct 5;17(10):e0274698. doi: 10.1371/journal.pone.0274698 (PMC9534387; doi:10.1371/journal.pone.0274698)

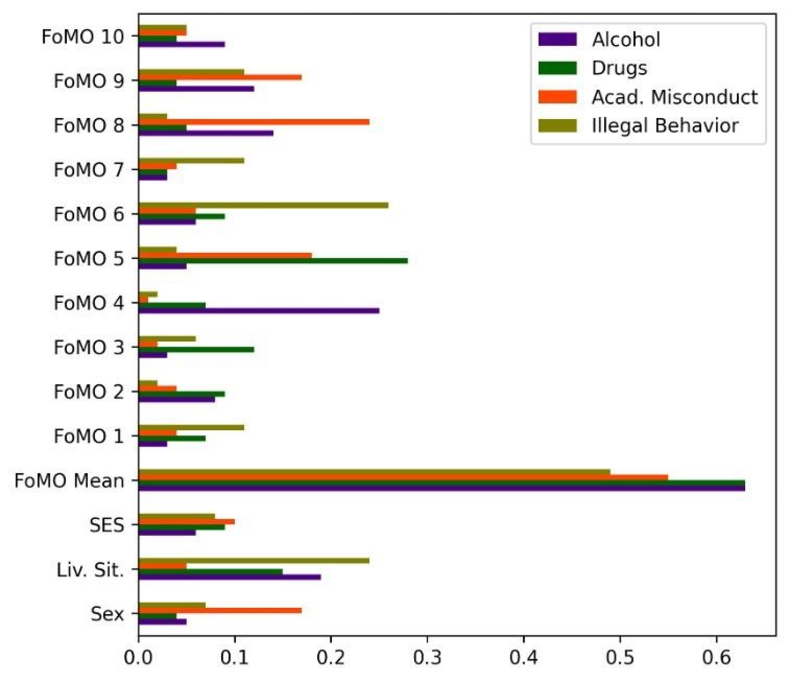

Supplement: S1 Fig — Mean values of feature importance scores obtained from machine learning models for both modeling scenarios considered, aggregate and individual. The aggregate case includes the metric FoMO Mean as a predictor, whereas the individual scenario uses the 10 FoMO items denoted FoMO 1 to FoMO 10 (but not FoMO Mean). In the aggregate case, FoMO Mean produces the highest importance scores among the predictors across all behavior domains. In the individual case, the scores of the FoMO items vary substantially across behavior domains. (TIF) [file pone.0274698.s001.tif]
