# Supplementary material for: College student Fear of Missing Out (FoMO) and maladaptive behavior: Traditional statistical modeling and predictive analysis using machine learning
Source: PLoS One. 2022 Oct 5;17(10):e0274698. doi: 10.1371/journal.pone.0274698 (PMC9534387; doi:10.1371/journal.pone.0274698)
Supplement: S1 File — This file reports the full results for all HLR models for all maladaptive behaviors tested. (DOCX) [file pone.0274698.s002.docx]

**SUPPLEMENTARY MATERIALS FOR**

**College Student Fear of Missing Out (FoMO) and Maladaptive Behavior: Traditional Statistical Modeling and Predictive Analysis using Machine Learning**

S1 File. Part 1 Hypothesis Testing Full Results.

| *Hierarchical Regression Analyses* | | | | | |  |
| --- | --- | --- | --- | --- | --- | --- |
| *Classroom Incivility* | | | | | | |
|  | *Predictor* | *b* | *t (468)* | *p* | *CI95%* | *ΔR2* |
| Model 1: |  |  |  |  |  |  |
| Step 1: | Socioeconomic Status | 0.025 | 0.761 | 0.447 | -0.039, 0.089 | 0.080 |
|  | FoMO | 0.171 | 6.258 | <.001 | 0.117, 0.225 |  |
| Model 2: |  |  |  |  |  |  |
| Step 1: | Socioeconomic Status | 0.032 | 0.980 | .327 | -0.032, 0.096 | 0.080 |
|  | FoMO | 0.355 | 3.292 | .001 | 0.143, 0.567 |  |
| Step 2: | FoMO X  Socioeconomic Status | -0.064 | -1.765 | .078 | -0.135, 0.007 | 0.006 |
| *Note*. Full model (*F*[3,468] = 14.758, *p* < .001, *R2* = .086). | | | | | | |
| *Classroom Incivility* | | | | | | |
|  | *Predictor* | *b* | *t (464)* | *p* | *CI95%* | *ΔR2* |
| Model 1: |  |  |  |  |  |  |
| Step 1: | Gender | -0.102 | -2.016 | .044 | -0.201, -0.003 | 0.087 |
|  | FoMO | 0.173 | 6.376 | <.001 | 0.120, 0.226 |  |
| Model 2: |  |  |  |  |  |  |
| Step 1: | Gender | -0.102 | -2.015 | .045 | -0.201, -0.003 | 0.087 |
|  | FoMO | 0.142 | 1.262 | .208 | -0.079, 0.364 |  |
| Step 2: | FoMO X Gender | 0.018 | 0.281 | .779 | -0.105, 0.140 | 0.000 |
| *Note*. Full model (*F*[3,468] = 14.917, *p* < .001, *R2* = .087). | | | | | | |
| *Classroom Incivility* | | | | | | |
|  | *Predictor* | *b* | *t (464)* | *p* | *CI95%* | *R2* |
| Model 1: |  |  |  |  |  |  |
| Step 1 | FoMO | 0.174 | 6.363 | <.001 | 0.120, 0.227 | 0.082 |
|  | Residence Hall^^[[1]](#footnote-1)^^ | 0.55 | 1.129 | .259 | -0.041, 0.150 |  |
|  | Off-Campus | 0.020 | 0.245 | .806 | -0.143, 0.184 |  |
|  | Other | 0.177 | 0.616 | .538 | -0.387, 0.741 |  |
| Model 2: |  |  |  |  |  |  |
| Step 1 | FoMO | 0.207 | 4.47 | < .001 | 0.116, 0.298 |  |
|  | Residence Hall | 0.141 | 1.024 | 0.306 | -0.130, 0.413 |  |
|  | Off-Campus | 0.324 | 1.423 | 0.155 | -0.123, 0.772 |  |
|  | Other | -0.163 | -0.243 | 0.808 | -1.478, 1.152 |  |
| Step 2 | FoMO X Residence Hall | -0.039 | -0.667 | 0.505 | -0.156, 0.077 | 0.005 |
|  | FoMO X Off-Campus | -0.144 | -1.431 | 0.153 | -0.342, 0.054 |  |
|  | FoMO X Other | 0.163 | 0.568 | 0.57 | -0.402, 0.728 |  |
| *Note*. Full model (F[7, 464] = 6.341, p < .001, R2 = .087 | | | | | | |

| *Hierarchical Regression Analyses* | | | | | |  |
| --- | --- | --- | --- | --- | --- | --- |
| *Plagiarism - in college* | | | | | | |
|  | *Predictor* | *b* | *t (468)* | *p* | *CI95%* | *ΔR2* |
| Model 1: |  |  |  |  |  |  |
| Step 1: | Socioeconomic Status | 0.012 | 0.231 | .817 | -0.093, 0.118 | 0.019 |
|  | FoMO | 0.132 | 2.934 | .004 | 0.044, 0.221 |  |
| Model 2: |  |  |  |  |  |  |
| Step 1: | Socioeconomic Status | 0.026 | 0.479 | .632 | -0.080, 0.132 | 0.019 |
|  | FoMO | 0.470 | 2.639 | .009 | 0.120, 0.820 |  |
| Step 2: | FoMO X  Socioeconomic Status | -0.117 | -1.959 | .051 | -0.234, 0.000 | 0.008 |
| *Note*. Full model (*F*[3,468] = 4.252, *p* = .006, *R2* = .027). | | | | | | |
| *Plagiarism - in college* | | | | | | |
|  | *Predictor* | *b* | *t (468)* | *p* | *CI95%* | *ΔR2* |
| Model 1: |  |  |  |  |  |  |
| Step 1: | Gender | -0.193 | -2.321 | .021 | -0.357, 0.030 | 0.030 |
|  | FoMO | 0.133 | 2.982 | .003 | 0.045, 0.221 |  |
| Model 2: |  |  |  |  |  |  |
| Step 1: | Gender | -0.193 | -2.320 | .021 | -0.357, -0.030 | 0.030 |
|  | FoMO | 0.025 | 0.137 | .891 | -0.339, 0.390 |  |
| Step 2: | FoMO X Gender | 0.062 | 0.599 | .550 | -0.141, 0.264 | 0.001 |
| *Note*. Full model (*F*[3,468] = 4.880, *p* = .002, *R2* = .030). | | | | | | |
| *Plagiarism - in college* | | | | | | |
|  | *Predictor* | *b* | *t (464)* | *p* | *CI95%* | *ΔR2* |
| Model 1: |  |  |  |  |  |  |
| Step 1 | FoMO | 0.136 | 3.042 | 0.002 | .048, .225 | 0.027 |
|  | Residence Hall | 0.038 | 0.477 | 0.634 | -.119, .195 |  |
|  | Off-Campus | 0.264 | 1.932 | 0.054 | -.004, .533 |  |
|  | Other | -0.261 | -0.553 | 0.581 | -1.189, 0.667 |  |
| Step 1 | FoMO | 0.136 | 3.042 | 0.002 | .048, .225 | 0.027 |
| Model 2: |  |  |  |  |  |  |
| Step 1 | FoMO | 0.05 | 0.656 | 0.512 | -.100, .200 |  |
|  | Residence Hall | -0.292 | -1.286 | 0.199 | -.738, .154 |  |
|  | Off-Campus | 0.175 | 0.468 | 0.64 | -.561, .912 |  |
|  | Other | -0.164 | -0.149 | 0.881 | -2.328, 1.999 |  |
| Step 2 | FoMO X Residence Hall | 0.151 | 1.555 | 0.121 | -.040, 342 | 0.005 |
|  | FoMO X Off-Campus | 0.038 | 0.229 | 0.819 | -.288, .364 |  |
|  | FoMO X Other | -0.05 | -0.106 | 0.916 | -.979, .879 |  |
| *Note*. Full model (F[7, 464] = 0.877, p = .453, R2 = .033). | | | | | | |

| *Hierarchical Regression Analyses* | | | | | |  |
| --- | --- | --- | --- | --- | --- | --- |
| *Cheating - in college* | | | | | | |
|  | *Predictor* | *b* | *t (468)* | *p* | *CI95%* | *ΔR2* |
| Model 1: |  |  |  |  |  |  |
| Step 1: | Socioeconomic Status | 0.049 | 0.611 | .541 | -0.108, 0.205 | 0.007 |
|  | FoMO | 0.107 | 1.606 | .109 | -0.024, 0.239 |  |
| Model 2: |  |  |  |  |  |  |
| Step 1: | Socioeconomic Status | 0.059 | 0.739 | .460 | -0.098, 0.217 | 0.007 |
|  | FoMO | 0.374 | 1.415 | .158 | -0.146, 0.894 |  |
| Step 2: | FoMO X  Socioeconomic Status | -0.092 | -1.043 | .298 | -0.267, 0.082 | 0.002 |
| *Note*. Full model (*F*[3,468] = 1.417, *p* = .237, *R2* = .009). | | | | | | |
| *Cheating - in college* | | | | | | |
|  | *Predictor* | *b* | *t (468)* | *p* | *CI95%* | *ΔR2* |
| Model 1: |  |  |  |  |  |  |
| Step 1: | Gender | -0.277 | -2.247 | .025 | -0.520, -0.035 | 0.016 |
|  | FoMO | 0.111 | 1.675 | .095 | -0.019, 0.241 |  |
| Model 2: |  |  |  |  |  |  |
| Step 1: | Gender | -0.278 | -2.250 | .025 | -0.520, -0.035 | 0.016 |
|  | FoMO | -0.197 | -0.718 | .473 | -0.737, 0.343 |  |
| Step 2: | FoMO X Gender | 0.176 | 1.156 | .248 | -0.123, 0.476 | 0.003 |
| *Note*. Full model (*F*[3,468] = 3.069, *p* = .028, *R2* = .019). | | | | | | |
| *Cheating - in college* | | | | | | |
|  | *Predictor* | *b* | *t (468)* | *p* | *CI95%* | *ΔR2* |
| Model 1: |  |  |  |  |  |  |
| Step 1: | FoMO | 0.119 | 1.795 | 0.073 | -.011, .249 | 0.025 |
|  | Residence Hall | 0.096 | 0.813 | 0.417 | -.136, .327 |  |
|  | Off-Campus | 0.606 | 3.002 | 0.003 | .209, 1.002 |  |
|  | Other | 0.171 | 0.246 | 0.806 | -1.197, 1.540 |  |
| Model 2: |  |  |  |  |  |  |
| Step 1 | FoMO | 0.254 | 2.267 | 0.024 | .034, .475 |  |
|  | Residence Hall | 0.537 | 1.606 | 0.109 | -.120, 1.193 |  |
|  | Off-Campus | 0.932 | 1.69 | 0.092 | -.152, 2.016 |  |
|  | Other | 2.596 | 1.602 | 0.11 | -.588, 5.781 |  |
| Step 2 | FoMO X Residence Hall | -0.202 | -1.408 | 0.16 | -.483, .080 | 0.009 |
|  | FoMO X Off-Campus | -0.149 | -0.612 | 0.541 | -.629, .330 |  |
|  | FoMO X Other | -1.148 | -1.65 | 0.1 | -2.517, .220 |  |
| *Note*. Full model (F[7, 464] = 2.303, p = .026, R2 = .034). | | | | | | |

| *Hierarchical Regression Analyses* | | | | | |  |
| --- | --- | --- | --- | --- | --- | --- |
| *Typical Weekly Alcohol* | | | | | | |
|  | *Predictor* | *b* | *t (468)* | *p* | *CI95%* | *ΔR2* |
| Model 1: |  |  |  |  |  |  |
| Step 1: | Socioeconomic Status | 0.534 | 1.161 | .246 | -0.369, 1.437 | 0.045 |
|  | FoMO | 1.718 | 4.453 | <.001 | 0.960, 2.476 |  |
| Model 2: |  |  |  |  |  |  |
| Step 1: | Socioeconomic Status | 0.583 | 1.258 | .209 | -0.328, 1.494 | 0.045 |
|  | FoMO | 2.954 | 1.935 | .054 | -0.046, 5.955 |  |
| Step 2: | FoMO X  Socioeconomic Status | -0.428 | -0.837 | .403 | -1.434, 0.577 | 0.001 |
| *Note*. Full model (*F*[3,468] = 7.672, *p* < .001, *R2* = .047). | | | | | | |
| *Typical Weekly Alcohol* | | | | | | |
|  | *Predictor* | *b* | *t (468)* | *p* | *CI95%* | *ΔR2* |
| Model 1: |  |  |  |  |  |  |
| Step 1: | Gender | -1.745 | -2.449 | .015 | -3.144, -0.345 | 0.055 |
|  | FoMO | 1.758 | 4.599 | <.001 | 1.007, 2.509 |  |
| Model 2: |  |  |  |  |  |  |
| Step 1: | Gender | -1.745 | -2.449 | .015 | -3.146, -0.345 | 0.055 |
|  | FoMO | 0.874 | 0.550 | .582 | -2.246, 3.993 |  |
| Step 2: | FoMO X Gender | 0.506 | 0.574 | .566 | -1.226, 2.237 | 0.001 |
| *Note*. Full model (*F*[3,468] = 9.159, *p* < .001, *R2* = .055). | | | | | | |
| *Typical Weekly Alcohol* | | | | | | |
|  | *Predictor* | *b* | *t (464)* | *p* | *CI95%* | *ΔR2* |
| Model 1: |  |  |  |  |  |  |
| Step 1: | FoMO | 1.804 | 4.732 | <0.001 | 1.055, 2.553 | 0.065 |
|  | Residence Hall | 1.703 | 2.507 | 0.013 | 0.368, 3.037 |  |
|  | Off-Campus | 3.382 | 2.909 | 0.004 | 1.097, 5.668 |  |
|  | Other | -0.011 | -0.003 | 0.998 | -7.895, 7.874 |  |
| Model 2: |  |  |  |  |  |  |
| Step 1 | FoMO | 0.731 | 1.131 | 0.259 | -0.539, 2.000 | 0.065 |
|  | Residence Hall | -2.246 | -1.168 | 0.243 | -6.026, 1.534 |  |
|  | Off-Campus | 1.057 | 0.333 | 0.739 | -5.182, 7.295 |  |
|  | Other | 4.758 | 0.510 | 0.610 | -13.569, 23.084 |  |
| Step 2 | FoMO X Residence Hall | 1.809 | 2.194 | 0.029 | 0.189, 3.428 | 0.011 |
|  | FoMO X Off-Campus | 1.058 | 0.753 | 0.452 | -1.701, 3.817 |  |
|  | FoMO X Other | -2.320 | -0.579 | 0.563 | -10.193, 5.553 |  |

| *Depressants* | | | | | | |
| --- | --- | --- | --- | --- | --- | --- |
|  | *Predictor* | *b* | *t (468)* | *p* | *CI95%* | *ΔR2* |
| Model 1: |  |  |  |  |  |  |
| Step 1: | Socioeconomic Status | -0.066 | -1.691 | .092 | -0.142, 0.011 | 0.032 |
|  | FoMO | 0.120 | 3.686 | <.001 | 0.056, 0.185 |  |
| Model 2: |  |  |  |  |  |  |
| Step 1: | Socioeconomic Status | -0.062 | -1.589 | .113 | -0.140, 0.015 | 0.032 |
|  | FoMO | 0.206 | 1.591 | .112 | -0.048, 0.460 |  |
| Step 2: | FoMO X  Socioeconomic Status | -0.030 | -0.682 | .495 | -0.115, 0.056 | 0.001 |
| *Note*. Full model (*F*[3,468] = 5.290, *p* = .001, *R2* = .033). | | | | | | |
| *Depressants* | | | | | | |
|  | *Predictor* | *b* | *t (468)* | *p* | *CI95%* | *ΔR2* |
| Model 1: |  |  |  |  |  |  |
| Step 1: | Gender | -0.101 | -1.674 | .095 | -0.221, 0.018 | 0.032 |
|  | FoMO | 0.115 | 3.542 | <.001 | 0.051, 0.179 |  |
| Model 2: |  |  |  |  |  |  |
| Step 1: | Gender | -0.101 | -1.672 | .095 | -0.221, 0.018 | 0.032 |
|  | FoMO | 0.124 | 0.916 | .360 | -0.142, 0.389 |  |
| Step 2: | FoMO X Gender | -0.005 | -0.065 | .948 | -0.152, 0.143 | 0.000 |
| *Note*. Full model (*F*[3,468] = 5.112, *p* = .002, *R2* = .032). | | | | | | |
| *Depressants* | | | | | | |
|  | *Predictor* | *b* | *t (468)* | *p* | *CI95%* | *ΔR2* |
| Model 1: |  |  |  |  |  |  |
| Step 1: | FoMO | 0.035 | 4.253 | < .001 | .019, .051 | 0.045 |
|  | Residence Hall | 0.02 | 1.364 | 0.173 | -.009, .048 |  |
|  | Off-Campus | 0.073 | 2.937 | 0.003 | .024, .122 |  |
|  | Other | -0.028 | -0.32 | 0.749 | -.196, .141 |  |
| Model 2: |  |  |  |  |  |  |
| Step 1 | FoMO | 0.038 | 2.704 | 0.007 | .010, .065 |  |
|  | Residence Hall | 0.035 | 0.844 | 0.399 | -.046, .116 |  |
|  | Off-Campus | 0.044 | 0.647 | 0.518 | -.090, .179 |  |
|  | Other | 0.052 | 0.258 | 0.797 | -.343, .447 |  |
| Step 2 | FoMO X Residence Hall | -0.007 | -0.391 | 0.696 | -.042, .028 | 0.002 |
|  | FoMO X Off-Campus | 0.014 | 0.465 | 0.642 | -.045, .074 |  |
|  | FoMO X Other | -0.038 | -0.436 | 0.663 | -.207, .132 |  |
| *Note*. Full model (F[7, 464] = 3.835, p < .001, R2 = .055). | | | | | | |

| *Hierarchical Regression Analyses* | | | | | |  |
| --- | --- | --- | --- | --- | --- | --- |
| *Stimulants* | | | | | | |
|  | *Predictor* | *b* | *t (468)* | *p* | *CI95%* | *ΔR2* |
| Model 1: |  |  |  |  |  |  |
| Step 1: | Socioeconomic Status | 0.011 | 0.191 | .849 | -0.105, 0.127 | 0.029 |
|  | FoMO | 0.183 | 3.689 | <.001 | 0.085, 0.280 |  |
| Model 2: |  |  |  |  |  |  |
| Step 1: | Socioeconomic Status | 0.011 | 0.179 | .858 | -0.106, 0.128 | 0.029 |
|  | FoMO | 0.167 | 0.852 | .395 | -0.219, 0.553 |  |
| Step 2: | FoMO X  Socioeconomic Status | 0.005 | 0.082 | .935 | -0.124, 0.135 | 0.000 |
| *Note*. Full model (*F*[3,468] = 4.624, *p* = .003, *R2* = .029). | | | | | | |
| *Stimulants* | | | | | | |
|  | *Predictor* | *b* | *t (468)* | *p* | *CI95%* | *ΔR2* |
| Model 1: |  |  |  |  |  |  |
| Step 1: | Gender | -0.200 | -2.190 | .029 | -0.380, -0.021 | 0.039 |
|  | FoMO | 0.184 | 3.738 | <.001 | 0.087, 0.280 |  |
| Model 2: |  |  |  |  |  |  |
| Step 1: | Gender | -0.201 | -2.198 | .028 | -0.380, -0.021 | 0.039 |
|  | FoMO | -0.164 | -0.809 | .419 | -0.564, 0.235 |  |
| Step 2: | FoMO X Gender | 0.199 | 1.763 | .079 | -0.023, 0.421 | 0.006 |
| *Note*. Full model (*F*[3,468] = 7.329, *p* < .001, *R2* = .045). | | | | | | |
| *Stimulants* | | | | | | |
|  | *Predictor* | *b* | *t (468)* | *p* | *CI95%* | *ΔR2* |
| Model 1: |  |  |  |  |  |  |
| Step 1: | FoMO | 0.02 | 3.712 | 0 | .009, .030 | 0.057 |
|  | Residence Hall | 0.008 | 0.804 | 0.422 | -.011, .026 |  |
|  | Off-Campus | 0.05 | 3.028 | 0.003 | .017, .082 |  |
|  | Other | 0.148 | 2.614 | 0.009 | .037, .259 |  |
| Model 2: |  |  |  |  |  |  |
| Step 1 | FoMO | 0.017 | 1.902 | 0.058 | -.001, .035 |  |
|  | Residence Hall | -0.004 | -0.131 | 0.896 | -.057, .050 |  |
|  | Off-Campus | 0.073 | 1.632 | 0.103 | -.015, .161 |  |
|  | Other | -0.036 | -0.277 | 0.782 | -.295, .222 |  |
| Step 2 | FoMO X Residence Hall | 0.005 | 0.443 | 0.658 | -.018, .028 | 0.006 |
|  | FoMO X Off-Campus | -0.011 | -0.579 | 0.563 | -.050, .027 |  |
|  | FoMO X Other | 0.088 | 1.548 | 0.122 | -.024, .199 |  |
| *Note*. Full model (F[7, 464] = 4.479, p < .001, R2 = .063). | | | | | | |

| *Hierarchical Regression Analyses* | | | | | |  |
| --- | --- | --- | --- | --- | --- | --- |
| *Cannabis* | | | | | | |
|  | *Predictor* | *b* | *t (468)* | *p* | *CI95%* | *ΔR2* |
| Model 1: |  |  |  |  |  |  |
| Step 1: | Socioeconomic Status | -0.097 | -1.337 | .182 | -0.240, 0.046 | 0.019 |
|  | FoMO | 0.173 | 2.835 | .005 | 0.053, 0.292 |  |
| Model 2: |  |  |  |  |  |  |
| Step 1: | Socioeconomic Status | -0.095 | -1.294 | .196 | -0.239, 0.049 | 0.019 |
|  | FoMO | 0.230 | 0.953 | .341 | -0.244, 0.704 |  |
| Step 2: | FoMO X  Socioeconomic Status | -0.020 | -0.246 | .806 | -0.179, 0.139 | 0.000 |
| *Note*. Full model (*F*[3,468] = 3.080, *p* = .027, *R2* = .019). | | | | | | |
| *Cannabis* | | | | | | |
|  | *Predictor* | *b* | *t (468)* | *p* | *CI95%* | *ΔR2* |
| Model 1: |  |  |  |  |  |  |
| Step 1: | Gender | 0.036 | 0.320 | .749 | -0.186, 0.259 | 0.016 |
|  | FoMO | 0.165 | 2.718 | .007 | 0.046, 0.284 |  |
| Model 2: |  |  |  |  |  |  |
| Step 1: | Gender | 0.036 | 0.320 | .749 | -0.186, 0.259 | 0.016 |
|  | FoMO | 0.149 | 0.589 | .556 | -0.347, 0.645 |  |
| Step 2: | FoMO X Gender | 0.009 | 0.067 | .947 | -0.266, 0.285 | 0.000 |
| *Note*. Full model (*F*[3,468] = 2.491, *p* = .060, *R2* = .016). | | | | | | |
| *Cannabis* | | | | | | |
|  | *Predictor* | *b* | *t (468)* | *p* | *CI95%* | *ΔR2* |
| Model 1: |  |  |  |  |  |  |
| Step 1 | FoMO | 0.175 | 2.948 | 0.003 | .058, .291 | 0.069 |
|  | Residence Hall | 0.474 | 4.492 | < .001 | .267, .682 |  |
|  | Off-Campus | 0.628 | 3.475 | 0.001 | .273, .983 |  |
|  | Other | 1.299 | 2.083 | 0.038 | .073, 2.524 |  |
| Model 2: |  |  |  |  |  |  |
| Step 1 | FoMO | 0.131 | 1.3 | 0.194 | -.067, .329 |  |
|  | Residence Hall | 0.253 | 0.845 | 0.399 | -.336, .843 |  |
|  | Off-Campus | 0.964 | 1.947 | 0.052 | -.009, 1.938 |  |
|  | Other | 0.596 | 0.41 | 0.682 | -2.263, 3.455 |  |
| Step 2 | FoMO X Residence Hall | 0.101 | 0.788 | 0.431 | -.151, .354 | 0.004 |
|  | FoMO X Off-Campus | -0.164 | -0.749 | 0.454 | -.595, .266 |  |
|  | FoMO X Other | 0.333 | 0.532 | 0.595 | -.896, 1.561 |  |
|  |  |  |  |  |  |  |
| *Note*. Full model (F[7, 464] = 5.244, p < .001, R2 = .073). | | | | | | |

| *Hierarchical Regression Analyses* | | | | | |  |
| --- | --- | --- | --- | --- | --- | --- |
| *Stealing - in college* | | | | | | |
|  | *Predictor* | *b* | *t (468)* | *p* | *CI95%* | *ΔR2* |
| Model 1: |  |  |  |  |  |  |
| Step 1: | Socioeconomic Status | -0.056 | -0.876 | .381 | -0.182, 0.070 | 0.031 |
|  | FoMO | 0.207 | 3.841 | <.001 | 0.101, 0.313 |  |
| Model 2: |  |  |  |  |  |  |
| Step 1: | Socioeconomic Status | -0.073 | -1.135 | .257 | -0.200, 0.054 | 0.031 |
|  | FoMO | -0.218 | -1.026 | .305 | -0.636, 0.199 |  |
| Step 2: | FoMO X  Socioeconomic Status | 0.147 | 2.068 | .039 | 0.007, 0.287 | 0.009 |
| *Note*. Full model (*F*[3,468] = 6.467, *p* < .001, *R2* = .040). | | | | | | |
| *Stealing - in college* | | | | | | |
|  | *Predictor* | *b* | *t (468)* | *p* | *CI95%* | *ΔR2* |
| Model 1: |  |  |  |  |  |  |
| Step 1: | Gender | -0.106 | -1.064 | .288 | -0.303, 0.090 | 0.032 |
|  | FoMO | 0.202 | 3.775 | <.001 | 0.097, 0.308 |  |
| Model 2: |  |  |  |  |  |  |
| Step 1: | Gender | -0.106 | -1.062 | .289 | -0.303, 0.090 | 0.032 |
|  | FoMO | 0.311 | 1.397 | .163 | -0.127, 0.749 |  |
| Step 2: | FoMO X Gender | -0.062 | -0.503 | .615 | -0.305, 0.181 | 0.001 |
| *Note*. Full model (*F*[3,468] = 5.209, *p* = .002, *R2* = .032). | | | | | | |
| *Stealing - in college* | | | | | | |
|  | *Predictor* | *b* | *t (468)* | *p* | *CI95%* | *ΔR2* |
| Model 1: |  |  |  |  |  |  |
| Step 1 | FoMO | 0.209 | 3.944 | <.001 | .105, .313 | 0.059 |
|  | Residence Hall | 0.271 | 2.871 | 0.004 | .086, .457 |  |
|  | Off-Campus | 0.511 | 3.16 | 0.002 | .193, .829 |  |
|  | Other | -0.311 | -0.557 | 0.577 | -1.408, .786 |  |
| Model 2: |  |  |  |  |  |  |
| Step 1 | FoMO | 0.25 | 2.768 | 0.006 | .072, .427 |  |
|  | Residence Hall | 0.442 | 1.644 | 0.101 | -.086, .971 |  |
|  | Off-Campus | 0.41 | 0.924 | 0.356 | -.462, 1.283 |  |
|  | Other | 0.218 | 0.167 | 0.868 | -2.345, 2.780 |  |
| Step 2 | FoMO X Residence Hall | -0.078 | -0.679 | 0.497 | -.305, .148 | 0.002 |
|  | FoMO X Off-Campus | 0.051 | 0.259 | 0.796 | -.335, .437 |  |
|  | FoMO X Other | -0.25 | -0.446 | 0.656 | -1.351, .851 |  |
| *Note*. Full model (F[7, 464] = 4.256, p < .001, R2 = .060). | | | | | | |

| *Hierarchical Regression Analyses* | | | | | |  |
| --- | --- | --- | --- | --- | --- | --- |
| *Giving Away Illegal Drugs - in college* | | | | | | |
|  | *Predictor* | *b* | *t (468)* | *p* | *CI95%* | *ΔR2* |
| Model 1: |  |  |  |  |  |  |
| Step 1: | Socioeconomic Status | 0.021 | 0.363 | .717 | -0.092, 0.133 | 0.021 |
|  | FoMO | 0.149 | 3.098 | .002 | 0.054, 0.243 |  |
| Model 2: |  |  |  |  |  |  |
| Step 1: | Socioeconomic Status | 0.026 | 0.450 | .653 | -0.087, 0.139 | 0.021 |
|  | FoMO | 0.279 | 1.465 | .144 | -0.095, 0.652 |  |
| Step 2: | FoMO X  Socioeconomic Status | -0.045 | -0.705 | .481 | -0.170, 0.080 | 0.001 |
| *Note*. Full model (*F*[3,468] = 3.504, *p* = .015, *R2* = .022). | | | | | | |
| *Giving Away Illegal Drugs - in college* | | | | | | |
|  | *Predictor* | *b* | *t (468)* | *p* | *CI95%* | *ΔR2* |
| Model 1: |  |  |  |  |  |  |
| Step 1: | Gender | -0.322 | -3.667 | <.001 | -0.495, -0.150 | 0.048 |
|  | FoMO | 0.150 | 3.184 | .002 | 0.058, 0.243 |  |
| Model 2: |  |  |  |  |  |  |
| Step 1: | Gender | -0.323 | -3.666 | <.001 | -0.495, -0.150 | 0.048 |
|  | FoMO | 0.039 | 0.200 | .841 | -0.346, 0.424 |  |
| Step 2: | FoMO X Gender | 0.063 | 0.584 | .560 | -0.150, 0.277 | 0.001 |
| *Note*. Full model (*F*[3,468] = 7.977, *p* < .001, *R2* = .049). | | | | | | |
| *Giving Away Illegal Drugs - in college* | | | | | | |
|  | *Predictor* | *b* | *t (468)* | *p* | *CI95%* | *ΔR2* |
| Model 1: |  |  |  |  |  |  |
| Step 1 | FoMO | 0.154 | 3.23 | 0.001 | 060, .248 | 0.031 |
|  | Residence Hall | 0.132 | 1.557 | 0.12 | -.035, .299 |  |
|  | Off-Campus | 0.289 | 1.989 | 0.047 | .003, .576 |  |
|  | Other | -0.214 | -0.425 | 0.671 | -1.201, .773 |  |
| Model 2: |  |  |  |  |  |  |
| Step 1 | FoMO | 0.202 | 2.483 | 0.013 | .042, .361 |  |
|  | Residence Hall | 0.252 | 1.043 | 0.298 | -.233, .728 |  |
|  | Off-Campus | 0.643 | 1.609 | 0.108 | -.142, 1.427 |  |
|  | Other | 0.214 | 0.183 | 0.855 | -2.091, 2.520 |  |
| Step 2 | FoMO X Residence Hall | -0.055 | -0.528 | 0.598 | -.258, .149 | 0.002 |
|  | FoMO X Off-Campus | -0.167 | -0.946 | 0.344 | -.514, .180 |  |
|  | FoMO X Other | -0.202 | -0.4 | 0.689 | -1.192, .789 |  |
| *Note*. Full model (F[7, 464] = 2.305, p = .026, R2 = .034). | | | | | | |

| *Hierarchical Regression Analyses* | | | | | |  |
| --- | --- | --- | --- | --- | --- | --- |
| *Giving Away Rx Drugs - in college* | | | | | | |
|  | *Predictor* | *b* | *t (468)* | *p* | *CI95%* | *ΔR2* |
| Model 1: |  |  |  |  |  |  |
| Step 1: | Socioeconomic Status | -0.010 | -0.411 | .681 | -0.060, 0.039 | 0.009 |
|  | FoMO | 0.045 | 2.091 | .037 | 0.003, 0.087 |  |
| Model 2: |  |  |  |  |  |  |
| Step 1: | Socioeconomic Status | -0.015 | -0.588 | .557 | -0.065, 0.035 | 0.009 |
|  | FoMO | -0.071 | -0.839 | .402 | -0.236, 0.095 |  |
| Step 2: | FoMO X  Socioeconomic Status | 0.040 | 1.414 | .158 | -0.016, 0.095 | 0.004 |
| *Note*. Full model (*F*[3,468] = 2.144, *p* = .094, *R2* = .014). | | | | | | |
| *Giving Away Rx Drugs - in college* | | | | | | |
|  | *Predictor* | *b* | *t (468)* | *p* | *CI95%* | *ΔR2* |
| Model 1: |  |  |  |  |  |  |
| Step 1: | Gender | -0.066 | -1.661 | .097 | -0.143, 0.012 | 0.015 |
|  | FoMO | 0.044 | 2.065 | .039 | 0.002, 0.085 |  |
| Model 2: |  |  |  |  |  |  |
| Step 1: | Gender | -0.066 | -1.666 | .096 | -0.143, 0.012 | 0.015 |
|  | FoMO | -0.080 | -0.908 | .364 | -0.252, 0.093 |  |
| Step 2: | FoMO X Gender | 0.071 | 1.449 | .148 | -0.025, 0.166 | 0.004 |
| *Note*. Full model (*F*[3,468] = 3.050, *p* = .028, *R2* = .019). | | | | | | |
| *Giving Away Rx Drugs - in college* | | | | | | |
|  | *Predictor* | *b* | *t (468)* | *p* | *CI95%* | *ΔR2* |
| Model 1: |  |  |  |  |  |  |
| Step 1 | FoMO | 0.043 | 2.036 | 0.042 | .002, .085 | 0.01 |
|  | Residence Hall | -0.015 | -0.385 | 0.7 | -.089, .060 |  |
|  | Off-Campus | -0.029 | -0.454 | 0.65 | -.157, .098 |  |
|  | Other | -0.076 | -0.338 | 0.735 | -.516, .365 |  |
| Model 2: |  |  |  |  |  |  |
| Step 1 | FoMO | 0.119 | 3.312 | 0.001 | .048, .189 |  |
|  | Residence Hall | 0.285 | 2.668 | 0.008 | .075, .495 |  |
|  | Off-Campus | -0.05 | -0.285 | 0.775 | -.397, .296 |  |
|  | Other | 0.181 | 0.349 | 0.727 | -.837, 1.199 |  |
| Step 2 | FoMO X Residence Hall | -0.137 | -2.999 | 0.003 | -.227, '-.047 | 0.023 |
|  | FoMO X Off-Campus | 0.014 | 0.183 | 0.855 | -.139, .168 |  |
|  | FoMO X Other | -0.119 | -0.534 | 0.594 | -.556, .318 |  |
| *Note*. Full model (F[7, 464] = 2.219, p = .032, R2 = .032). | | | | | | |

*Note.* These tables report the full results for all HLR models for all maladaptive behaviors tested.

1. All living situations (i.e., residence halls, off-campus [not with parents], other) are coded 1 and compared to living with parents which was coded zero. [↑](#footnote-ref-1)
