# Supplementary material for: College student Fear of Missing Out (FoMO) and maladaptive behavior: Traditional statistical modeling and predictive analysis using machine learning
Source: PLoS One. 2022 Oct 5;17(10):e0274698. doi: 10.1371/journal.pone.0274698 (PMC9534387; doi:10.1371/journal.pone.0274698)
Supplement: S2 File — (DOCX) [file pone.0274698.s003.docx]

**SUPPLEMENTARY MATERIALS FOR**

**College Student Fear of Missing Out (FoMO) and Maladaptive Behavior: Traditional Statistical Modeling and Predictive Analysis using Machine Learning**

**S2 File. More Machine Learning Approach Info:**

Decision trees are supervised machine learning classifiers that filter data in the likeness of trees: Roots to branches to leaf nodes. Using if-then sorting, decision trees classify data into progressively smaller sub-categories. Random forest classifiers are an ensemble of individual decision trees working together, to provide the best predictive model based on majority group consensus.

SVM algorithms are especially useful and achieve greater predictive accuracy when the classes are not linearly separable. It is important to note that while SVM is technically a linear classifier, the use of the Radial Basis Function (RBF) kernel allows data to be classified when the relationship is nonlinear.

Logistic regression classifiers stem from traditional statistics in which the probability of the default class is modeled using a sigmoid function. Probability values are then converted into either of the two class labels using a thresholding approach.

In addition to just offering predictive value from the input variables we provide; machine learning techniques can be applied to perform dimensionality reduction of the data. Two techniques we use are Recursive Feature Elimination (RFE) and Principal Component Analysis (PCA). RFE selects a subset of features from the data that maximizes predictive power, while PCA creates a linear combination of a smaller number of features. To explore the merit of dimensionality reduction techniques we also applied RFE and PCA in combination with a random forest classifier.
