# Supplementary material for: College student Fear of Missing Out (FoMO) and maladaptive behavior: Traditional statistical modeling and predictive analysis using machine learning
Source: PLoS One. 2022 Oct 5;17(10):e0274698. doi: 10.1371/journal.pone.0274698 (PMC9534387; doi:10.1371/journal.pone.0274698)
Supplement: S3 File — Decision trees for the classification of academic misconduct, alcohol, and illegal behavior based on the FoMO aggregate scenario. Starting at the root node, an example is evaluated in a sequential manner down the tree based on the conditions in the decision nodes. A classification is made according to the end node reached (blue denotes a positive prediction and light orange a negative prediction). (DOCX) [file pone.0274698.s004.docx]

**SUPPLEMENTARY MATERIALS FOR**

**College Student Fear of Missing Out (FoMO) and Maladaptive Behavior: Traditional Statistical Modeling and Predictive Analysis using Machine Learning**

SF1. Part 2 Decision Tree Output for Academic Misconduct, Alcohol Use, and Illegal Behaviors.


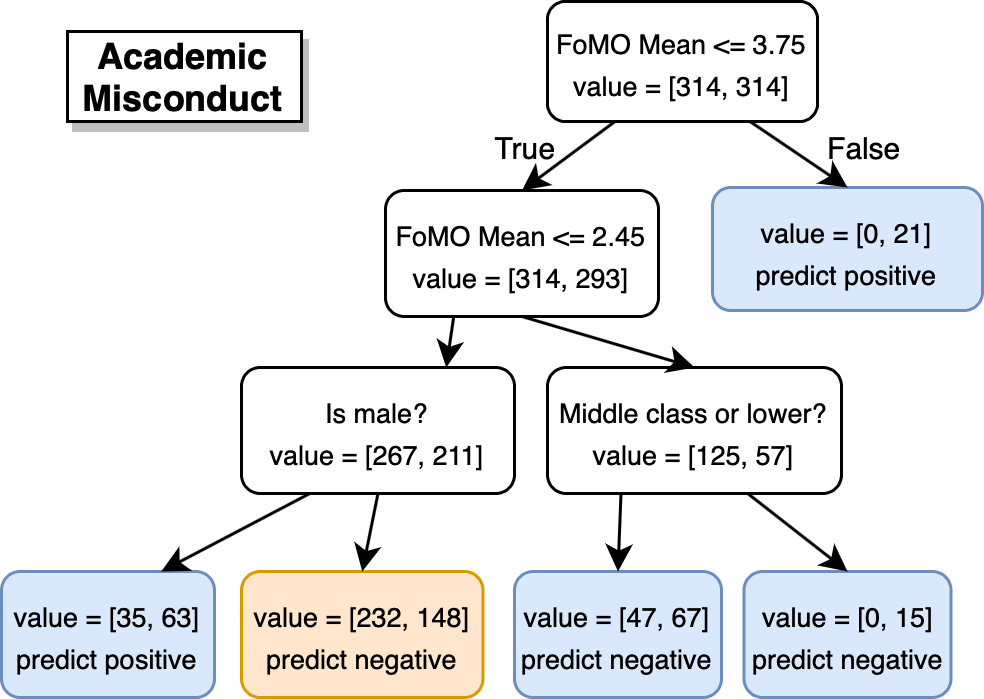


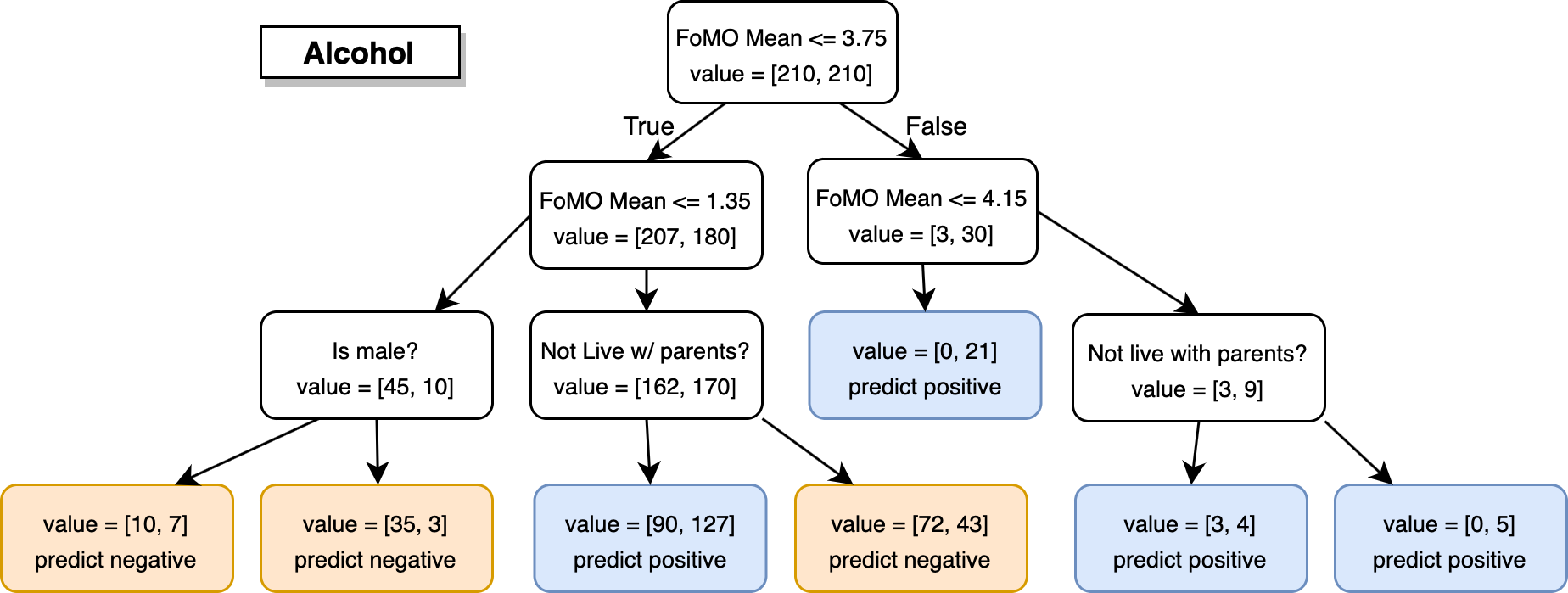


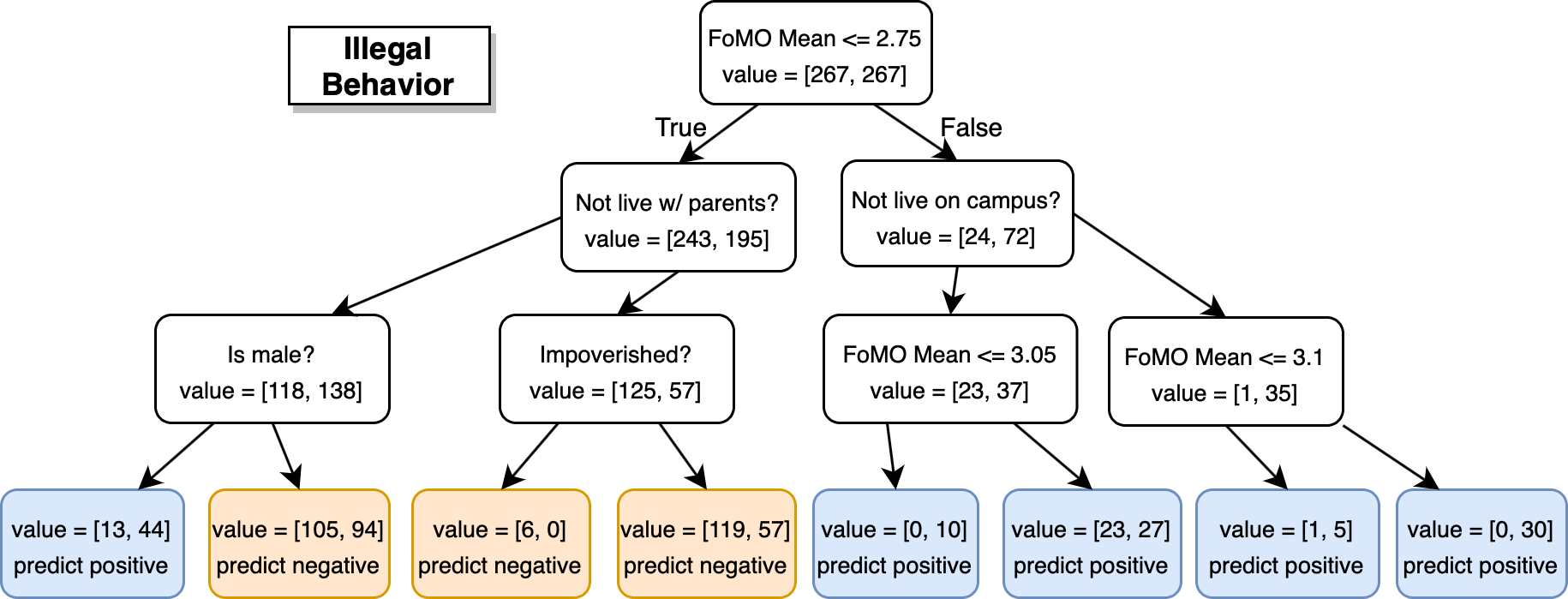


Note. Decision trees for the classification of academic misconduct, alcohol, and illegal behavior based on the FoMO aggregate scenario. Starting at the root node, an example is evaluated in a sequential manner down the tree based on the conditions in the decision nodes. A classification is made according to the end node reached (blue denotes a positive prediction and light orange a negative prediction).
