# Supplementary material for: College student Fear of Missing Out (FoMO) and maladaptive behavior: Traditional statistical modeling and predictive analysis using machine learning
Source: PLoS One. 2022 Oct 5;17(10):e0274698. doi: 10.1371/journal.pone.0274698 (PMC9534387; doi:10.1371/journal.pone.0274698)
Supplement: Attachment — Submitted filename: Review20220524.pdf [file pone.0274698.s005.pdf]

Overall, according to the reviewing guidelines offered by PLOS ONE, I find this manuscript includes a fair treatment of previous literature in this area, well articulated hypotheses, valid and appropriate data, adequate modeling details, thorough reporting, and thoughtful conclusions.

I applaud the authors for so clearly categorizing and articulating their individual hypotheses. This is excellent analysis and reporting practice and limits so-called “fishing expeditions” when such hypotheses are planned prior to looking at the data and contribute to reproducibility in science. The authors also report all regression statistics, estimates, and p-values, which further contributes to transparency and reproducibility.

The authors provide a clear and concise explanation of the differences between statistical inference and machine learning prediction. Furthermore, the descriptions of test/training sets and k-fold cross-validation were concise and useful and will assist a reader unfamiliar with these concepts. Later in the manuscript, the authors also describe the dual function of some machine learning approaches in providing both feature selection/importance as well as predicted values.

Recommendations:

1. The hypotheses are dropped into the text without introduction. I recommend including a sentence somewhere in the beginning of Part 1 to the effect of, “Six total hypotheses were tested regarding the relationship between FoMO and academic misconduct, substance use, and illegal behaviors…” to help orient the reader.
2. Recommend removing the term “significant” from the stated hypotheses unless the authors wish to outline the parameters under which the association coefficients for each variable will be deemed “clinically significant.” Otherwise, “significant” here is assumed to pertain to the statistical test and this is implied in the hypothesis testing itself.
3. The stated hypotheses specify a direction of effect (“positively”) and a ranking of effect (“strongest”) and neither of these were necessarily directly tested. The hypotheses were tested in the regression context that utilizes two-sided tests, as is usually done, and so I recommend rephrasing the hypotheses to reflect this. Generally, this is backwards – adjusting the hypothesis to fit the test, but in this case, it seems as though this is a point of clarification and specificity of language, not of post-hoc “adjustment.” I recommend altering the working to something along the lines of, “FoMO will be associated with illegal behavior” and “Covariates X, Y, and Z will moderate the above association” to avoid implying one-directional tests that are not actually conducted.
4. For all 3 categories of tests, the second hypothesis includes a sex association, but while the cited literature supports the hypotheses regarding socioeconomic status and living situation, I didn’t note any literature supporting this hypothesis for male sex.
5. It is not immediately clear how the 6 stated hypotheses align with and/or are integrated into the research questions listed on page 6, lines 215-218.
6. The “Part 1” and “Part 2” headings in the 2.2 and 2.3 sections are confusing, perhaps they are misplaced?
7. Recommend rephrasing Page 6, lines 211-212 from, “This work expands our understanding of college student FoMO while contributing to the recent shift toward utilizing multiple statistical approaches” to something along the lines of, “This work leverages multiple complimentary statistical and machine learning approaches to expand

our understanding of college student FoMO," as implementing both inferential and predictive methods together is arguably not a recent development.

8. The justification for categorizing the maladaptive behavior measures on page 8 is difficult to follow. The statements, "Furthermore, the current approach was data driven. Hence, it was preferable to use the more efficient binary classifications so long as a dimensional approach was not more accurate" seems to imply that the authors tested multiple approaches (binary and dimensional) but this doesn't seem to have been done? I might recommend simply removing those statements altogether and simply stating that as a initial analysis, considering that binary classifications are typically those clinically utilized, a binary classification approach was adopted. Then I recommend adding some sentences in the limitations/future work section to suggest that exploring the fully dimensionality of the behavioral measurements may be of future interest.
9. The results on pages 14-16 are difficult to parse in the text. I recommend moving the F statistics and degrees of freedom to the table and removing those and the beta estimates and p-values from the text – so long as these values are reported in the table, they do not need also to be repeated in the text. It may also be useful to revisit editing this section for conciseness. Furthermore, there is no reference to this table in the text.
10. Recommend reiterating for the reader in the paragraph beginning on page 18 (line numbers not available here) that the "Aggregate" FoMO value is the mean score while the "Individual" is the sum of items.
11. In contrast to item 3, noted above, the results for the machine learning section are sparse, with only tables and figures and very little text explaining these. I recommend adding some sentences summarizing the findings in the table. Some of the performance metrics information from the discussion on page 23 in particular might be better suited moved to the results section.
12. The in-text references to tables on page 18 are misnumbered
13. Recommend adding an overall caption for the tables in the supplement
14. One of the supplemental tables is blank
15. I highly recommend that the authors consider making the analytical code for the machine learning approaches publicly available via GitHub or some other platform. Given that machine learning is not broadly, openly acceptable as a standard analysis approach by everyone in the social, behavioral, and biological sciences, making code openly available contributes to transparency and reproducibility in science and builds trust among our collaborators.
